# Role of metallic core for the stability of virus-like particles in strongly coupled electrostatics

**DOI:** 10.1038/s41598-019-39930-8

**Published:** 2019-03-07

**Authors:** Leili Javidpour, Anže Lošdorfer Božič, Rudolf Podgornik, Ali Naji

**Affiliations:** 1 School of Physics, Institute for Research in Fundamental Sciences (IPM), Tehran, 19395-5531 Iran; 2 Department of Theoretical Physics, Jožef Stefan Institute, SI-1000 Ljubljana, Slovenia; 3 School of Physical Sciences and Kavli Institute for Theoretical Sciences, University of Chinese Academy of Sciences, Beijing, 100049 China; 4 CAS Key Laboratory of Soft Matter Physics, Institute of Physics, Chinese Academy of Sciences (CAS), Beijing, 100190 China; 5 Department of Physics, Faculty of Mathematics and Physics, University of Ljubljana, SI-1000 Ljubljana, Slovenia

## Abstract

Electrostatic interactions play important roles in the formation and stability of viruses and virus-like particles (VLPs) through processes that often involve added, or naturally occurring, multivalent ions. Here, we investigate the electrostatic or osmotic pressure acting on the proteinaceous shell of a generic model of VLPs, comprising a charged outer shell and a metallic nanoparticle core, coated by a charged layer and bathed in an aqueous electrolyte solution. Motivated by the recent studies accentuating the role of multivalent ions for the stability of VLPs, we focus on the effects of multivalent cations and anions in an otherwise monovalent ionic solution. We perform extensive Monte-Carlo simulations based on appropriate Coulombic interactions that consistently take into account the effects of salt screening, the dielectric polarization of the metallic core, and the strong-coupling electrostatics due to multivalent ions. We specifically study the intricate roles these factors play in the electrostatic stability of the model VLPs. It is shown that while the insertion of a metallic nanoparticle by itself can produce negative, inward-directed, pressure on the outer shell, addition of only a small amount of multivalent counterions can robustly engender negative pressures, enhancing the VLP stability across a wide range of values for the system parameters.

## Introduction

Encapsidation of non-biological cargo in viral proteinaceous capsids has attracted a lot of interest in recent years, connected with their role as (noninfectious) virus-like particles (VLPs) in applications such as gene transfer, drug delivery^1^, engineering of modern vaccine platforms^2,3^, as well as in biomedical imaging and therapeutics applications^4–7^. Encapsidation of metallic nanoparticles such as gold and iron-oxide cores^8^ in viral capsids has extensively been studied using experimental methodology^8–12^, and (to a lesser extent) using theoretical and computer simulation models^13,14^. Nanoparticle encapsidation is typically done through origin-of-assembly templating or polymer templating^4^. In the first scenario, the nanoparticle core is decorated by an origin-of-assembly site that initiates the binding of the coat proteins and drives the self-assembly around the core. In the second scenario, the nanoparticle core is decorated with negatively charged polymers intended to mimic the effects due to the negative charge of the native viral cargo, which is the (highly negatively charged) nucleic-acid (DNA or RNA) genome.

The interactions between the viral capsid and its native genomic cargo can include both non-specific and highly specific interactions, depending on the type of the virus. This consequently influences whether the encapsidation of artificial cargo can be achieved without help from nucleic acids, or whether oligonucleotides of a given length and specific sequence are needed for proper assembly^1,10,11,15–17^. A prime example of a virus that can serve in a variety of functional nanoparticle assemblies is the brome mosaic virus (BMV)^18^, while other ssRNA plant viruses, such as red clover necrotic mottle virus (RCNMV)^10,11^ and cowpea chlorotic mottle virus (CCMV)^17^ have also been used as nanoparticle containers. The capsids of these viruses usually carry hypotopal protein N-terminal tails with a high positive charge, which, in many cases, bind with the genome at least partially through non-specific electrostatic interactions^1^. Thus, nanoparticles decorated with negatively charged polymers may in principle mimic the electrostatic behavior of the genome and initiate the self-assembly of the viral capsid around the artificial core.

The chemical and physical properties of the non-biological VLP core play a major role in determining the efficiency of the formation of the proteinaceous shell around it, as well as in determining the VLP’s overall stability and electrostatic properties^9^. Recent experiments were successful in decoupling the role of charge and size of the encapsulated cargo in the assembly of VLPs^15^. It was observed that there is a critical charge density of the core below which the VLPs do not form, even if the core can otherwise fit well into the cavity of the proteinaceous shell and the total charge on the core is sufficient to completely neutralize the positively charged N-tails of a complete viral capsid.

The role of the bathing ionic solutions for the electrostatic stability of viruses and VLPs has also been studied in recent years^13^, primarily based on the mean-field Poisson-Boltzmann (PB) framework^19,20^. It is however known from more recent developments^21–26^ that even a small amount of multivalent ions in the system can dramatically shift the governing electrostatic paradigm, from that described by the PB theory to a conceptually different paradigm known as the strong-coupling or, in its generalized form in the case of an ionic mixture, the dressed multivalent-ion theory^26^. This latter situation is believed to occur in many biologically relevant examples^21,27,28^. The counter-intuitive like-charge attraction is a major manifestation of strong-coupling interactions mediated by multivalent ions between macromolecular surfaces^21–26^, and it is considered to underlie exotic phenomena such as formation of large DNA condensates^29–33^ and large bundles of microtubules^34^ and F-actin^35,36^. Multivalent ions are also known to play a key role in dense DNA packaging in viruses and nano-capsids^37–44^ although their effects are still not completely understood.

Motivated by these developments and the relevance of electrostatic interactions for the stability of the VLP formation, we investigate the effects of multivalent cations and anions in an otherwise monovalent ionic bathing solution, in which a model VLP is formed. We formulate a theoretical approach that can take into account polarization of the metallic nanoparticle core in the presence of ionic screening as well as the strong-coupling electrostatics generated by the presence of solution multivalent ions. In particular, we address the combined effect of the dielectric polarization, or dielectric images, arising from the discontinuous jump of the dielectric constant at the metallic core surface, and the salt polarization, or salt images, arising from the inhomogeneous distribution of the salt ions that are excluded from the core region (see also the recent work in ref.^45^). The description of electrostatic interactions between the components of the ionic solution and the VLP charge distribution is then used in Monte-Carlo simulations to compute the net, equilibrium, electrostatic or osmotic pressure exerted on the proteinaceous shell of the VLP in the presence and absence of the metallic nanoparticle core. We specifically address the change of sign in this pressure as a result of the strong-coupling electrostatics generated by multivalent ions in the bathing solution.

## Model and Methods

### Model: Geometry and basic features

**Figure 1:**
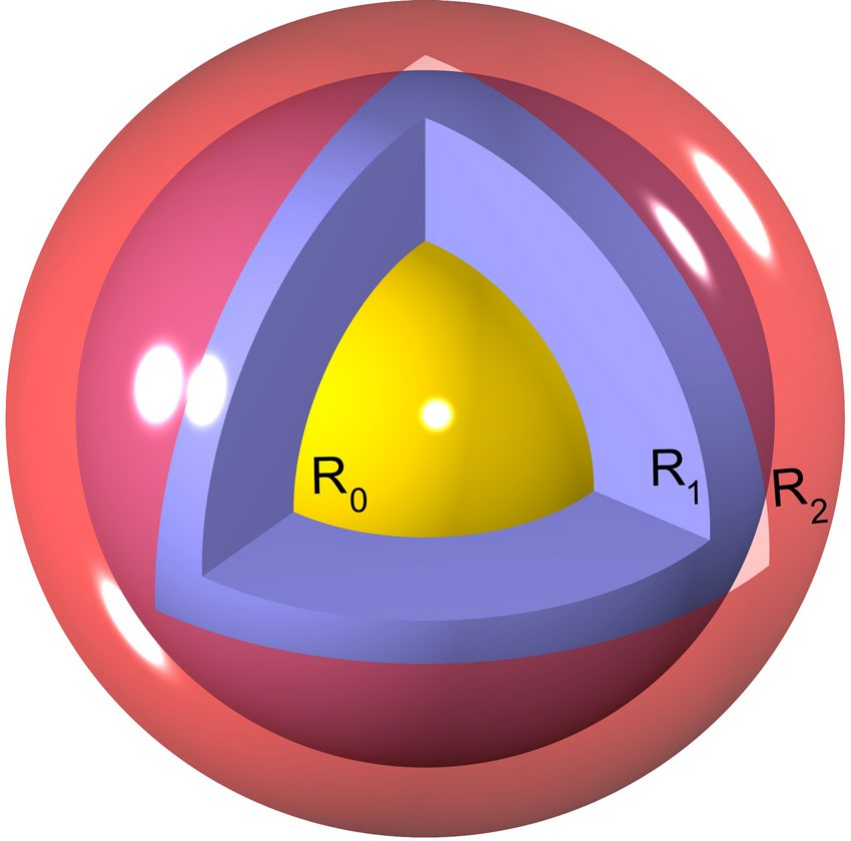
(Sketch of the VLP model system). Schematic view of a model VLP comprising an electroneutral metallic (yellow) core of radius *R*_0_, coated by a (blue) layer of outer radius *R*_1_ and surface charge density *σ*_1_, encapsidated in a charged (red) shell of radius *R*_2_ and surface charge density *σ*_2_ (see the text for details). For the sake of illustration, parts of the outer shell and the coating layer are removed in the schematic picture to show the core region.

We model the metallic nanoparticle (NP) core as an ideally polarizable and electrostatically neutral sphere of radius *R*_0_, decorated with a coating layer of outer radius *R*_1_ (representing, e.g., a polymeric layer such as the negatively charged polyethylene glycol used to cover encapsidated gold NPs^9,15,16^). The coating layer in general bears a surface charge density of *σ*_1_*e*_0_, where *e*_0_ is the elementary charge. The enclosing capsid will likewise be treated as a spherical shell of radius *R*_2_ and surface charge density *σ*_2_*e*_0_ (see Fig. 1). For simplicity, we shall refer to the charge distribution of the coating layer and that of the capsid as the “inner” and “outer” charged shells. While the metallic core is assumed to be strictly impermeable, the capsid and the coating layer are assumed to be permeable to water molecules and monovalent solution ions, which can thus be present within the whole region *r* > *R*_0_. The (generally larger) multivalent ions are assumed to permeate inside the shell but not within the coating layer with *r* < *R*_1_. As such, we take the region outside the metallic core as a medium of uniform dielectric constant, *ε*. The bathing solution is assumed to contain a base monovalent salt (1:1) and an additional asymmetric (*q*:1) salt of bulk concentrations *n*_0_ and *c*_0_, respectively.

When multivalent ions have a relatively large valency *q* (typically |*q*| > 2, as exemplified by polyamines^46,47^, e.g., tri- and tetravalent spermidine and spermine, and ionic complexes, e.g., trivalent cobalt hexammine^48^), the ionic mixture can no longer be treated using traditional mean-field approaches (applicable to monovalent ions)^21^, nor using standard strong-coupling methods (applicable to multivalent counterion-only solutions)^22–25^, as one must concurrently account for the weak- and strong-coupling nature of interactions mediated by mono- and multivalent ions, respectively^26^. The dressed multivalent-ion approach^49^ provides one such framework in the case of highly asymmetric ionic mixtures and in a relatively broad range of salt concentrations (with the concentration of multivalent ions, *c*_0_, being typically less than few tens of mM). Having been successfully tested against simulations^50–53^ and recent experiments^54^ within its predicted regime of validity, it can offer considerable nontrivial simplification in the study of highly asymmetric ionic mixtures by systematically integrating out the monovalent degrees of freedom, enabling one to focus exclusively on the interactions of multivalent ions and the fixed macromolecular surface charges^26^. Thus, while the former (monovalent species) will be treated implicitly on the linearized mean-field, or Debye-Hückel (DH) level, the latter (multivalent ions and fixed charges) will be treated explicitly. Analytical results will also be attainable by further approximation (known as the limiting dressed multivalent-ion theory), treating the multivalent-ion correlations with the fixed charges on the leading *single-particle* level, following the virial-expansion scheme adopted within the strong-coupling electrostatics^22,23,26^. The single-particle characteristics of strong-coupling phenomena, arising due to multivalent ions near charged surfaces^22,23^, and the collective, mean-field, characteristics of monovalent ions, producing Debye screening effects, are thus both incorporated within a single framework.

The Green’s function associated with electrostatic interactions in the described model, *G*(***r***, ***r***′), represents the effective interaction potential of two test unit charges placed at positions ***r*** and ***r***′ outside the metallic core, which is centered at the origin; it can be derived as (see the Supplementary Information)

1 $$\begin{array}{c}G({\bf{r}},{\bf{r}}{\boldsymbol{^{\prime} }})=\frac{{{\rm{e}}}^{-\kappa |{\bf{r}}-{\bf{r}}{\boldsymbol{^{\prime} }}|}}{4\pi \varepsilon {\varepsilon }_{0}|{\bf{r}}-{\bf{r}}{\boldsymbol{^{\prime} }}|}+\frac{{\kappa }^{2}{R}_{0}\,{{\rm{e}}}^{2\kappa {R}_{0}}}{4\pi \varepsilon {\varepsilon }_{0}(1+\kappa {R}_{0})}{k}_{0}(\kappa r){k}_{0}(\kappa r^{\prime} )\\ \,\,\,\,-\frac{\kappa }{4\pi \varepsilon {\varepsilon }_{0}}\sum _{l=0}^{\infty }\,(2l+1)\frac{{i}_{l}(\kappa {R}_{0})}{{k}_{l}(\kappa {R}_{0})}{k}_{l}(\kappa r^{\prime} ){k}_{l}(\kappa r){P}_{l}(\cos \,\vartheta ),\end{array}$$

where *i*_*l*_(⋅) and *k*_*l*_(⋅) are modified spherical Bessel functions of the first and second kind, respectively, *P*_*l*_(·) are Legendre polynomials, and we have defined *r* = |***r***|, *r*′ = |***r***′|, and *ϑ* as the angle between ***r*** and ***r***′. The first term in Eq. (1) incorporates the direct screened Coulomb (or DH) interaction between the test charges, with the inverse screening length *κ* defined through ${\kappa }^{2}=4\pi {\ell }_{{\rm{B}}}(2{n}_{0}+|q|{c}_{0})$, where ${\ell }_{{\rm{B}}}={e}_{0}^{2}/(4\pi \varepsilon {\varepsilon }_{0}{k}_{{\rm{B}}}T)$ is the Bjerrum length and 2*n*_0_ + |*q*|*c*_0_ is the total bulk concentration of monovalent ions. The second and third terms in Eq. (1) together give the contributions from the polarization effects. These contributions stem from (i) the induced polarization (“dielectric image”) charges produced by the test charges within the metallic NP core, and (ii) the induced polarization (“salt image”) charges produced because of the exclusion of the surrounding polarizable (and globally neutral), monovalent, ionic solution from the core region; the latter would be absent if the monovalent screening solution was present everywhere. It is however important to note that these two types of polarization effects are intrinsically entangled and enter in both the second and the third terms; also that the image charges in the metallic core cannot in general be conceived as individual Kelvin images^49–51^.

### Configurational hamiltonian and pressure

The Green’s function (1) can be used to construct the configurational Hamiltonian of any given arrangements of *N* multivalent ions (labeled by subscripts *i*, *j* = 1, …, *N* for their positions {***r***_*i*_}) as

2 $$H=\sum _{i=1}^{N}\,{U}_{ii}+\sum _{i > j=1}^{N}\,{U}_{ij}+\sum _{i=1}^{N}\,{U}_{i}^{\sigma }+{U}^{\sigma \sigma },$$

where *U*_*ii*_ is the self-energy of the *i*th multivalent ion, giving its self-interaction with its own image charges; *U*_*ij*_ is the contribution due to direct screened Coulomb interaction between distinct multivalent ions *i* and *j* and the cross interactions between these ions and their respective image charges; ${U}_{i}^{\sigma }$ is the contribution due to interactions between the *i*th multivalent ion *i* and the two charged (inner and outer) shells, to be subscripted by *α*, *β* = 1, 2 for their radius and surface charge density; and *U*^*σσ*^ is the contribution due to the interaction between the two charged shells. The image contributions to the ion-shell and shell-shell interactions are systematically included in ${U}_{i}^{\sigma }$ and *U*^*σσ*^. These contributions can explicitly be calculated as (see the Supplementary Information)

3 $$\frac{{U}_{ii}}{{k}_{{\rm{B}}}T}=-\,\frac{1}{2}{q}^{2}{\ell }_{{\rm{B}}}\kappa \sum _{l=0}^{\infty }\,(2l+1)\frac{{i}_{l}(\kappa {R}_{0})}{{k}_{l}(\kappa {R}_{0})}{k}_{l}^{2}(\kappa {r}_{i})+{q}^{2}{\ell }_{{\rm{B}}}\frac{{\kappa }^{2}{R}_{0}\,{{\rm{e}}}^{2\kappa {R}_{0}}}{2(1+\kappa {R}_{0})}{k}_{0}^{2}(\kappa {r}_{i}),$$

4 $$\begin{array}{l}\frac{{U}_{ij}}{{k}_{{\rm{B}}}T}={q}^{2}{\ell }_{{\rm{B}}}\frac{{{\rm{e}}}^{-\kappa |{{\bf{r}}}_{i}-{{\bf{r}}}_{j}|}}{|{{\bf{r}}}_{i}-{{\bf{r}}}_{j}|}+{q}^{2}{\ell }_{{\rm{B}}}\frac{{\kappa }^{2}{R}_{0}\,{{\rm{e}}}^{2\kappa {R}_{0}}}{\mathrm{(1}+\kappa {R}_{0})}{k}_{0}(\kappa {r}_{i}){k}_{0}(\kappa {r}_{j})\\ \,\,\,-{q}^{2}{\ell }_{{\rm{B}}}\kappa \sum _{l=0}^{\infty }\,(2l+1)\frac{{i}_{l}(\kappa {R}_{0})}{{k}_{l}(\kappa {R}_{0})}{k}_{l}(\kappa {r}_{i}){k}_{l}(\kappa {r}_{j}){P}_{l}(\cos \,{\vartheta }_{ij}),\end{array}$$

5 $$\frac{{U}_{i}^{\sigma }}{{k}_{{\rm{B}}}T}=\frac{2\pi q{\ell }_{{\rm{B}}}}{\kappa {r}_{i}}\sum _{\alpha =1}^{2}\,{\sigma }_{\alpha }{R}_{\alpha }[({{\rm{e}}}^{-\kappa |{r}_{i}-{R}_{\alpha }|}-{{\rm{e}}}^{-\kappa ({r}_{i}+{R}_{\alpha })})+{{\rm{e}}}^{-\kappa ({r}_{i}+{R}_{\alpha })}(1+{{\rm{e}}}^{2\kappa {R}_{0}}\frac{\kappa {R}_{0}-1}{\kappa {R}_{0}+1})],$$

6 $$\frac{{U}^{\sigma \sigma }}{{k}_{{\rm{B}}}T}=\frac{4{\pi }^{2}{\ell }_{{\rm{B}}}}{\kappa }\sum _{\alpha ,\beta =1}^{2}\,{\sigma }_{\alpha }{\sigma }_{\beta }{R}_{\alpha }{R}_{\beta }[({{\rm{e}}}^{-\kappa |{R}_{\alpha }-{R}_{\beta }|}-{{\rm{e}}}^{-\kappa |{R}_{\alpha }+{R}_{\beta }|})+{{\rm{e}}}^{-\kappa ({R}_{\alpha }+{R}_{\beta })}(1+{{\rm{e}}}^{2\kappa {R}_{0}}\frac{\kappa {R}_{0}-1}{\kappa {R}_{0}+1})],$$

where *r*_*i*_ = |***r***_*i*_|, *r*_*j*_ = |***r***_*j*_|, and *ϑ*_*ij*_ is the angle between ***r***_*i*_ and ***r***_*j*_.

In what follows, we shall focus on the net pressure, *P*, acting on the virus-like (outer) shell due to the combined effect of screened Coulomb interactions between fixed charges on the outer and inner shells and their interactions with the multivalent ions, as expressed in the configurational Hamiltonian, Eq. (2). We shall further examine how the dielectric image charges due to the metallic core influence the net pressure on the outer shell. This latter quantity can be computed from our simulations using the relation

7 $$P=-{(\frac{\partial \bar{H}}{\partial {V}_{2}})}_{{Q}_{2}},$$

where the bar represents the numerically evaluated (thermal) average over different equilibrium configurations of multivalent ions, ${V}_{2}=4\pi {R}_{2}^{3}/3$ is the outer shell volume, and the partial derivative is taken by fixing all system parameters except the surface density of the outer shell, *σ*_2_; indeed, it is the total charge of the outer shell, ${Q}_{2}=4\pi {R}_{2}^{2}{\sigma }_{2}$, that must be kept fixed. This is explicitly indicated in Eq. (7) and is related to the fact that the total number of capsomer charges is assumed to be chemically fixed and does not vary, unlike the surface charge density, *σ*_2_, that varies as the radius is changed. Needless to say that Eq. (7) gives the net thermodynamic pressure, which can equivalently be expressed also as the derivative of the free energy of the system with respect to *V*_2_; *P* involves the osmotic as well as the electrostatic Maxwell stress contributions and can be interpreted as the difference in pressure between the outside and inside of the outer shell. It can also be decomposed conveniently as

8 $$P={P}_{DH}+{P}_{q},$$

where *P*_*DH*_ is the baseline pressure acting on the outer shell in the absence of multivalent ions and *P*_*q*_ is the pressure contribution explicitly stemming from the interactions of the charge distributions of the shells with the multivalent ions, fully incorporating the effects due to their respective salt/dielectric images, as produced by the presence of the metallic core (thus, *P*_*q*_ = 0 when *q* = 0). The baseline pressure can be written as

9 $${P}_{DH}={P}_{{\sigma }_{2}{\sigma }_{2}}+{P}_{{\sigma }_{2}{\sigma }_{1}},$$

where ${P}_{{\sigma }_{2}{\sigma }_{2}} > 0$ is the (outward-directed) pressure component arising from the self-energy of the outer shell, and ${P}_{{\sigma }_{2}{\sigma }_{1}}$ is the pressure component arising from the interaction of the outer shell charge with the fixed charge on the inner shell, both systematically accounting for the salt/dielectric image effects due to the metallic core. The two can be obtained explicitly as

10 $${P}_{{\sigma }_{2}{\sigma }_{2}}=\frac{{\sigma }_{2}^{2}{e}_{0}^{2}}{2\varepsilon {\varepsilon }_{0}}\{\frac{1}{\kappa {R}_{2}}+{{\rm{e}}}^{-2\kappa ({R}_{2}-{R}_{0})}(\frac{\kappa {R}_{0}-1}{\kappa {R}_{0}+1})(1+\frac{1}{\kappa {R}_{2}})\},$$

11 $${P}_{{\sigma }_{2}{\sigma }_{1}}=\frac{{\sigma }_{2}{\sigma }_{1}{e}_{0}^{2}}{2\varepsilon {\varepsilon }_{0}}(\frac{{R}_{1}}{{R}_{2}})\{{{\rm{e}}}^{-\kappa ({R}_{2}-{R}_{1})}+{{\rm{e}}}^{-\kappa ({R}_{1}+{R}_{2}-2{R}_{0})}(\frac{\kappa {R}_{0}-1}{\kappa {R}_{0}+1})\}(1+\frac{1}{\kappa {R}_{2}}).$$

The multivalent-ion pressure *P*_*q*_ can be written as

12 $${P}_{q}=-\,\frac{q{\sigma }_{2}{e}_{0}^{2}}{8\pi \varepsilon {\varepsilon }_{0}\kappa {R}_{2}^{2}}\sum _{i=1}^{N}\overline{{\rm{\Pi }}({{\bf{r}}}_{i})},$$

where we have

13 $${\rm{\Pi }}({{\bf{r}}}_{i})=\frac{{{\rm{e}}}^{-\kappa |{r}_{i}-{R}_{2}|}}{{r}_{i}}(\kappa {R}_{2}\,{\rm{sgn}}\,({r}_{i}-{R}_{2})-1)-\frac{{{\rm{e}}}^{-\kappa ({r}_{i}+{R}_{2}-2{R}_{0})}}{{r}_{i}}(1+\kappa {R}_{2})(\frac{\kappa {R}_{0}-1}{\kappa {R}_{0}+1}),$$

where sgn(⋅) is the sign function.

We shall also discuss later the reference case of a model VLP with no NP core included in it. This case helps discern the effects of image charges produced by the NP core from other factors.

The expressions for *P*_*DH*_ and *P*_*q*_ in the presence and absence of the metallic NP core are discussed in further detail in the Supplementary Information.

### Simulation details

The configurational Hamiltonian, Eqs (2–6), can be used with appropriately designed Monte Carlo (MC) simulations to calculate equilibrium properties of the model VLP immersed in an asymmetric ionic mixture. The VLP is placed at the center of a cubic simulation box of lateral size 2(*R*_2_ + 8*κ*^−1^) with periodic boundary conditions. We are interested in relatively large values of *κ* (see Section 2.4), with the corresponding Debye screening lengths being consistently smaller than the VLP size and the simulation box size. Therefore, Coulomb interactions in the system are sufficiently short-ranged to enable a minimum-image convention for their calculation, without recourse to infinite summation schemes due to the assumed periodic boundary conditions^55^. As noted in Section 2.1, we follow the dressed multivalent-ion approach as described and tested previously^26,49–54^, by treating the monovalent ions implicitly (on a linearized DH level, with prescribed values for their bulk concentration or, equivalently, for their bulk inverse screening length, *κ*) and treating the multivalent ions explicitly (as spherical ions of finite radius, *a*; we take *a* = 0.15 nm, which is however of no particular relevance in our study, as we are focused on very dilute multivalent-salt regimes). Because of the screened nature of the Coulomb interactions in the present approach, the number of multivalent ions does *not* need to be fixed by imposing an electroneutrality condition on the *explicit* charges, which would have implied that the sum of the total charge of explicit multivalent ions and those of the inner and outer shells vanish; any net explicit charge in the system is in fact neutralized by the background monovalent ions for which an ionic bulk is *implicitly* assumed.

Since *κ* is taken to be sufficiently large (Section 2.4), finite-box-size effects are efficiently eliminated, ensuring that the simulated multivalent-ion density profiles always exhibit a relatively wide plateau-like region of constant density, *c*_0_, further away from the central VLP (not shown here; see, however, refs^52,53^). This region indeed acts as the ionic ‘bulk’, surrounding the VLP; it sets in at radial distances typically around a couple of Debye screening lengths away from the outer shell of the VLP and extends all the way to the bounding simulation box. Our simulations are in fact designed such as to generate the bulk condition within the simulation box and, as such, reproduce a physical situation equivalent to a grand-canonical one (see below). When placed in the context of colloidal cell models, our simulation box can thus be viewed as the unit cell of a dilute suspension of identical VLPs in a bathing ionic bulk in the salt-dominated regime^56^, with vanishing Donnan potential (note that the net electrostatic potential diminishes over the bulk region and on approach to the bounding simulation box); this latter point also ensures that the standard DH linearization scheme used for monovalent ions in the present context remains consistent with the observations made in ref.^56^.

In the simulations, we use a fixed number of explicit multivalent ions, *N*, in the simulation box, with *N* being adjusted suitably such as to produce equilibrium states with the desired value, *c*_0_, of the bulk concentration of explicit multivalent ions. Note that *N* is not a priori known and, even for a prescribed value of *c*_0_, it can vary depending on the other system parameters. To determine *N* for any given set of parameter values, we use the efficient iterative algorithm introduced by us in refs^52,53^. It utilizes a series of individual canonical simulations that iteratively estimate the desired value of *N* based on a linear interpolation scheme and, in only a few (typically around five) iterations, converge to a final simulation, which reproduces the bulk concentration *c*_0_ to within a relative error of 0.1%. This method provides an alternative approach to a grand-canonical simulation with a set bulk concentration or chemical potential of multivalent ions (see the Supplementary Information for further details).

The calculation of the contributions to the Hamiltonian (2) involve infinite summation series through Eqs (3) and (4). These summations include spherical Bessel functions, whose values are initially tabulated by evaluating them over a cubic mesh (lattice) of points in the space defined by the variables {*r*_*i*_, *r*_*j*_ > *r*_*i*_, cos *ϑ*_*ij*_}; the values of the spherical Bessel functions on off-lattice positions are obtained by tricubic interpolation^57^. The mesh is sufficiently refined such that the maximum relative error of the tabulation/interpolation scheme (as compared with the direct evaluation of off-lattice values) is of the order 10^−3^. The mentioned infinite summation series converge rapidly and are thus calculated using series cutoffs chosen such that the maximum relative truncation error will be of the order 10^−7^. The aforementioned errors are smaller than the typical (sampling) error bars of the simulations that are obtained using block-averaging methods^58^ to be of relative magnitude of at most 2%. All the simulations in this work run for 6 × 10^7^ MC steps per particle with the first 10^7^ steps used for equilibration purposes.

### Choice of parameter values

Our model parameters include the radius *R*_0_ of the NP core, the inner and outer shell radii *R*_1_ and *R*_2_ and their surface charge densities *σ*_1_ and *σ*_2_, respectively, the multivalent-ion charge valency *q*, the inverse screening length *κ* (supplemented by the ionic densities *n*_0_ and *c*_0_), and the solvent dielectric constant, which we shall equate to that of water at room temperature, *ε* = 80. Our primary goal is to investigate the generic electrostatic properties of the model VLP with a metallic core in the presence of (positively and negatively charged) multivalent ions and, as such, structural details that may be present in actual experimental systems are largely ignored (see Section 4). In order to bring out the salient roles of image charges and multivalent ions, especially in the net pressure they produce on the capsid (outer shell), we explore typical regions of the parameter space by fixing the values of system parameters that are of less immediacy to our analysis, i.e., the radii of the NP core and the two charged shells and the charge density of the capsid *σ*_2_, while we vary the other parameters.

In experiments with plant viruses (such as BMV, RCNMV, or CCMV) encapsidating a single coated gold NP^9–11,15–17^, the NP core radius typically ranges from 3–12 nm with a coating layer of thickness around 2 nm^15^. The size of the assembled viral capsid naturally corresponds with the size of the core; for instance, a sufficiently large core will lead to the assembly of wild-type BMV with the capsid triangulation number ${\mathscr{T}}=3$, while a smaller core will lead to an assembly of a smaller ${\mathscr{T}}=1$ capsid^9^. To provide an estimate of typical surface charge densities on the assembled capsids, we again take the example of BMV capsid^15^, which has a typical total charge of $Q=540{\mathscr{T}}{e}_{0}$. For smaller (${\mathscr{T}}=1$) capsids as, for instance, obtained by NP cores of radius *R*_0_ = 3 nm^9^, one can take the typical values of *R*_1_ = 5 nm^15^ and *R*_2_ = 6 nm^16^, giving an effective surface charge density of ${\sigma }_{2}\simeq 1.2$ nm^−2^ for the outer shell. Without loss of generality, we fix these typical numerical values for the most part in our numerical simulations and vary *σ*_1_, *κ* and, in some cases, *R*_1_ − *R*_0_.

The surface charge density of the coating layer, *σ*_1_, is varied in a wide range of values from −3 nm^−2^ up to +5 nm^−2^, covering also realistic values used or estimated in previous studies^14,15^. The inverse screening length is varied in the range *κ* = 0.5 − 2 nm^−1^ for three different cases of multivalent ion valencies, *q* = + 2, *q* = + 4 and *q* = −4. We fix the concentration of multivalent ions as *c*_0_ = 5 mM for divalent cations, and *c*_0_ = 1 mM for tetravalent cations and anions. Hence, for instance, the numerical value of *κ* = 0.78 nm^−1^ corresponds to monovalent ion concentration of ${n}_{0}\simeq 53$ mM in the case of divalent cations, and ${n}_{0}\simeq 56$ mM in the case of tetravalent cations/anions. These numerical values fall consistently within the experimentally accessible ranges of values^15^. The number of explicit multivalent ions in our simulations varies depending on the system parameters (as only their bulk concentration, *c*_0_, is controlled), with their typical numbers obtained as follows: For divalent (tetravalent) cations, the equilibrium number of particles in the final round of simulated (canonical) iterations varies between 88 (17) and 175 (68) as *σ*_1_ is varied between 0 and −2 nm^−2^. For tetravalent anions, their number varies between 55 and 541 as *σ*_1_ is increased from −2 nm^−2^ up to 5 nm^−2^.

Though some of the numerical values listed above are adopted directly from the BMV experiments, other ranges of parameter values corresponding to other NPs and other viral capsids (such as CCMV)^59^ can be equally well addressed with our generic electrostatic model of VLPs. Needless to say that our models has an obvious symmetry with respect to sign inversions *q* → −*q* and *σ*_1, 2_ → −*σ*_1, 2_.

## Results and Discussion

### Pressure components: Cations vs anions

**Figure 2:**
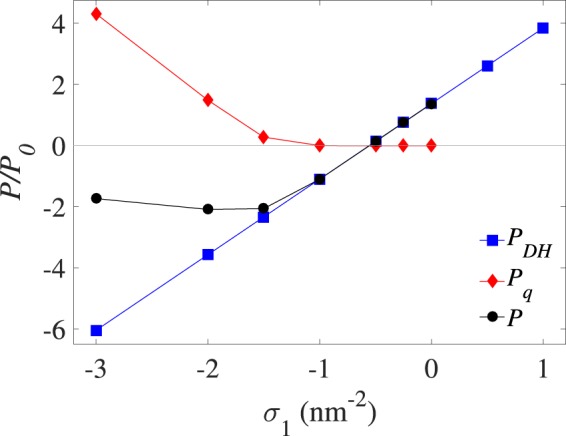
(Pressure vs σ_1_ for *q* = +2). Rescaled net pressure, *P*, acting on outer shell of the model VLP, and its different components, *P*_*DH*_ and *P*_*q*_, plotted as a function of the surface charge density of the NP coating layer (inner shell) for divalent cations (*q* = +2) at fixed values of the outer shell surface charge density *σ*_2_ = +1.2 nm^−2^ and radius *R*_2_ = 6 nm, the NP radius *R*_0_ = 3 nm, the coating layer thickness 2 nm (*R*_1_ = 5 nm), and the inverse Debye screening length $\kappa \simeq 0.78$ nm^−1^. Also, we have ${P}_{0}=1{k}_{{\rm{B}}}T/{{\rm{nm}}}^{3}\simeq 41$ atm.

In Fig. 2, we show our simulation results for the net pressure *P* on the outer virus-like shell, and its components *P*_*DH*_ and *P*_*q*_, as defined in Section 2.2, as a function of the coating surface charge density of the metallic NP core (or the inner shell charge density), *σ*_1_. Here, we have used divalent cations (*q* = 2) with the typical choices of values for fixed parameters as noted in Section 2.4; i.e., *σ*_2_ = 1.2 nm^−2^, *κ* = 0.78 nm^−1^ (with fixed *c*_0_ = 5 mM), *R*_0_ = 3 nm, *R*_1_ = 5 nm and *R*_2_ = 6 nm. In the plots hereafter we rescaled the pressure values with ${P}_{0}=1{k}_{{\rm{B}}}T/{{\rm{nm}}}^{3}\simeq 41$ atm.

As seen in the figure, the baseline pressure *P*_*DH*_ (blue squares) acting on the outer shell in the absence of multivalent ions increases linearly with *σ*_1_, which is in accordance with Eq. (11). *P*_*DH*_ changes sign from negative (inward-directed pressure on the outer shell) to positive values (outward-directed pressure) as *σ*_1_ is increased above ${\sigma }_{1}^{\ast }\simeq -0.55$ nm^−2^. The change of sign in *P*_*DH*_ occurs at a negative value of *σ*_1_ because of our choice of a positive outer shell charge density *σ*_2_ > 0; that is, the positive self-pressure of the outer shell ${P}_{{\sigma }_{2}{\sigma }_{2}}$ can be balanced by the negative inter-shell pressure component ${P}_{{\sigma }_{2}{\sigma }_{1}}$ (giving the inward-directed pull of the outer shell by the inner shell) only if the two shells are oppositely charged; see also Eq. (9).

Another point to be noted is that the pressure component due to divalent cations, *P*_*q*_ (red diamonds), vanishes for ${\sigma }_{1} > {\sigma }_{1}^{\ast \ast }\simeq -\,1$ nm^−2^, where the net pressure (black circles) equals the base pressure, $P\simeq {P}_{DH}$. *P*_*q*_ is nonzero only for ${\sigma }_{1} < {\sigma }_{1}^{\ast \ast }\simeq -\,1$ nm^−2^, where it takes sizably large (outward-directed pressure) values, partially balancing the negative (inward-directed) pressure due to *P*_*DH*_, or more accurately, ${P}_{{\sigma }_{2}{\sigma }_{1}}$; hence, producing a non-monotonic behavior in the net pressure, *P*, as a function of *σ*_1_ (Fig. 2). The reported net pressure in this latter regime of coating surface charge densities is thus a direct consequence of the inter-shell attraction ${P}_{{\sigma }_{2}{\sigma }_{1}}$, giving net values of around $P\simeq -40$ to −80 atm in actual units. This implies an electrostatically favorable situation for the formation and stability of the resulting VLP for ${\sigma }_{1} < {\sigma }_{1}^{\ast \ast }$, in contrast to the opposite scenario, which is predicted to hold in the regime ${\sigma }_{1} > {\sigma }_{1}^{\ast }$, where the net pressure becomes positive. The conclusion that a minimum negative value of |*σ*_1_| is required to enable formation of stable VLPs with divalent cations is generally consistent with recent findings in the BMV context^15^, where the BMV encapsidation of a coated gold NP is reported to occur only for *σ*_1_ < −2 nm^−2^, roughly corresponding to the location of the minimum net pressure (black circles) in Fig. 2.

**Figure 3:**
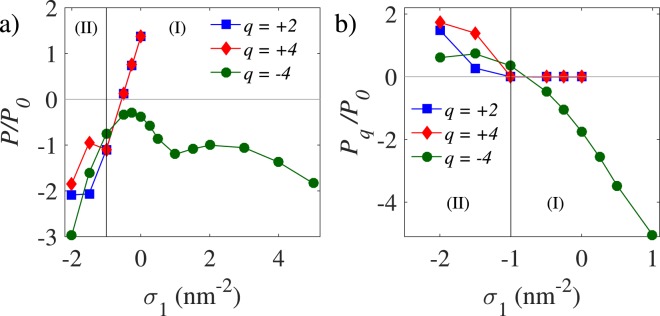
(Pressure vs σ_1_ for different q). Same as Fig. 2 but shown are (**a**) the net pressure and (**b**) the pressure contribution stemming from multivalent ions, *P*_*q*_, for three different values of *q*. The vertical lines show the coating layer charge density ${\sigma }_{1}^{\ast \ast }$, as introduced in the text.

The net pressure on the outer shell shows qualitatively similar behavior for varying cation charge valency (compare *q* = 2 and 4 in Fig. 3a) with significant deviations occurring only at sufficiently large magnitudes of (negative) *σ*_1_. However, a remarkably different behavior is observed if we use anionic multivalent ions, e.g., *q* = −4 (Fig. 3a). This is an interesting case as, in contrast to the case of cations, multivalent anions are electrostatically repelled from the inner NP coating layer (*σ*_1_ < 0), while they are attracted more strongly to the outer shell (*σ*_2_ > 0) as we shall discuss later in this section. The net pressure on the outer shell, *P*, becomes negative for multivalent anions (green circles in Fig. 3a) across the whole range of *σ*_1_ plotted in the figure. Thus, while in region (II) in the figure (${\sigma }_{1} < {\sigma }_{1}^{\ast \ast }$), one can obtain negative net pressure in both cases of multivalent cations and multivalent anions (even though *P*_*q*_ remains positive in either case in region (II); see Fig. 3b), only multivalent anions can produce a negative net pressure in region (I) (${\sigma }_{1} > {\sigma }_{1}^{\ast \ast }$). A more detailed comparison between the two exemplary cases with *q* = 2 and *q* = −4 ions will be given in Sections 3.3 and 3.4. Here, it is important to note that different mechanisms are at work in regions I and II, and also for cationic vs anionic multivalent ions. While, as noted above, the resulting negative net pressure in region (II) can be understood as indicating a dominant inter-shell attraction component, ${P}_{{\sigma }_{2}{\sigma }_{1}}$, regardless of the multivalent ion charge, the dependence of the net pressure on *q* in region (I) can be elucidated by examining the accumulation of multivalent ions within the VLP, as we shall do next.

### Multivalent-ion accumulation within VLP

**Figure 4:**
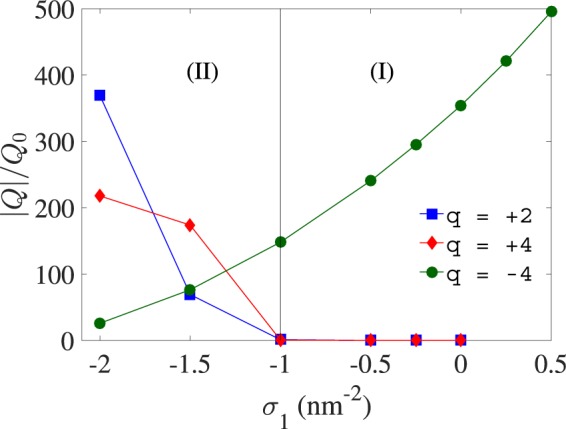
(Total multivalent-ion charge inside the VLP). Absolute value of the total multivalent-ion charge, |*Q*|, accumulated within inter-shell space of the VLP, *R*_1_ < *r* < *R*_2_, plotted as a function of the surface charge density of the NP coating layer for three different values of *q*. The results are rescaled with the characteristic charge ${Q}_{0}=(4\pi /3)({R}_{2}^{3}-{R}_{1}^{3})|q|{e}_{0}{c}_{0}$. See Fig. 2 for other parameters.

**Figure 5:**
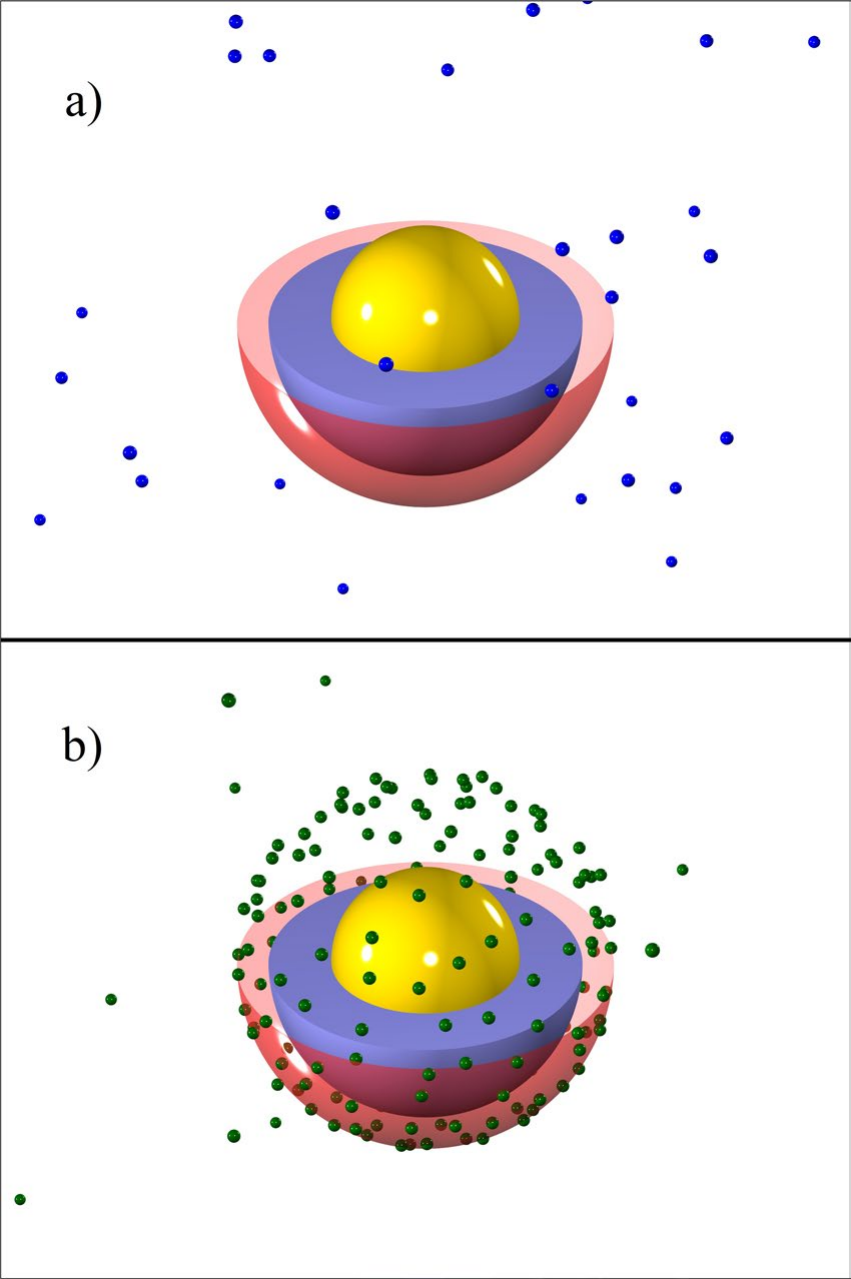
(Snapshots from the simulations). Simulation snapshot showing positions of multivalent ions in and outside the model VLP for (**a**) divalent cations (shown as small blue spheres) and (**b**) tetravalent anions (shown as small green spheres) for fixed *σ*_1_ = 0 and other parameter values given in Fig. 2. For the sake of illustration, the upper half of the outer shell (shown schematically as a red transparent sphere) and the NP coating layer (shown as a thick blue shell) are removed in the picture, while the core metallic NP is shown in whole (yellow sphere).

The absolute value of the total multivalent-ion charge, |*Q*|, accumulated within the inter-shell space of the VLP, *R*_1_ < *r* < *R*_2_, can directly be measured from our simulations. We rescale the accumulated charge with the characteristic charge ${Q}_{0}=(4\pi \mathrm{/3})({R}_{2}^{3}-{R}_{1}^{3})|q|{e}_{0}{c}_{0}$. The results, plotted in Fig. 4 as a function of *σ*_1_, clearly show that, while multivalent anions exhibit a smoothly decreasing degree of accumulation within the VLP by decreasing *σ*_1_ from positive to negative values, multivalent cations exhibit a complete depletion from within the VLP in the parameter region (I) (giving $|Q|\simeq 0$), followed by a sharp increase in |*Q*| as one enters region (II) by decreasing *σ*_1_ (see also simulation snapshots in Fig. 5). Although these behaviors can generally be understood based on the net charge of the VLP, i.e., the sum of the inner and outer shell charges, which becomes positive (negative) in region (I) (region (II)), the detailed behavior of *Q* is determined by the combined effect of the three interaction terms (3)–(5) and vary depending on the precise choice of system parameters.

The behavior of accumulated multivalent-ion charge in Fig. 4 also provides a better insight into the behavior of *P*_*q*_ in Fig. 3b. The near complete depletion of multivalent cations from within the VLP in region (I) clearly explains why ${P}_{q}\simeq 0$ and, hence, $P\simeq {P}_{DH}$, in the mentioned parameter region. Also, the stronger accumulation of multivalent cations within the VLP by decreasing *σ*_1_ in region (II) (making *σ*_1_ more negative; Fig. 4) shows that the positive *P*_*q*_ (blue and red symbols in Fig. 3b) results from the stronger repulsion these ions impart on the outer shell, which is also positively charged (formally, this repulsion is embedded in Eq. (5)).

In the case of multivalent anions, the positive *P*_*q*_ in region (II) (green circles in Fig. 3b) results from the image charges of these ions in the metallic NP core that will be positively charged; hence, producing the only source of repulsion on the outer shell (as embedded in Eq. (3)) that may stem from the multivalent ions in this case. Another distinct aspect of multivalent anions is that the pressure component *P*_*q*_ changes sign to take negative values of large magnitude in region (I), engendering also a large negative net pressure *P* on the outer shell, as noted before (Fig. 3a). We shall return to the underlying cause of this large negative pressure in Section 3.4.

We conclude this section by emphasizing that the change of sign in the pressure component *P*_*q*_ in going from region (II) to region (I) is essential in maintaining a negative net pressure on the outer shell across a broad range of values for *σ*_1_ in the case of multivalent anions. Our results thus also suggest that multivalent anions present a more robust case (in contrast to multivalent cations) in stabilizing the VLP, irrespective of the sign and magnitude of the surface charge density of the NP coating layer.

### Image charge effects

The effects due to the image charges on the net pressure, *P*, can be assessed by comparing the results obtained in the case of a VLP containing a hard, metallic NP core with those obtained in an equivalent model in which the sources of interfacial dielectric jump or inhomogeneous salt ion distribution are eliminated; that is, one in which the dielectric constant of the core is set equal to that of the aqueous solution, *ε* = 80, while the coating charge density, *σ*_1_, is kept fixed, and the monovalent salt (screening) ions are allowed to permeate within the core region (*r* < *R*_0_) as well. The cases with and without the metallic NP core, as described, are labeled in the following plots by “NP” and “no NP”, respectively. In the simulations of the “no NP” case, the dielectric and salt image eliminations are done by using only the free-space part of the Green’s function, i.e., by keeping the first term of Eq. (1) and omitting the other two terms. In either case, the multivalent ions remain restricted to the region *r* > *R*_1_.

**Figure 6:**
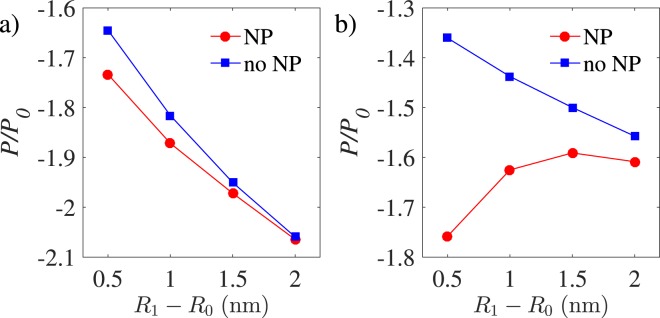
(Pressure vs the coating layer thickness). Rescaled net pressure is plotted as a function the coating layer thickness, *R*_1_ − *R*_0_, for (**a**) *q* = + 2 and (**b**) *q* = −4, and for fixed *σ*_1_ = −1.5 nm^−2^ and *R*_2_ − *R*_1_ = 1 nm. Other parameters are as in Fig. 2. See the text for details on the designations “NP” and “no NP” in the legends.

The image charge effects turn out to be small for divalent cations (*q* = 2) for the parameters chosen in Fig. 6a, while they turn out to be significant for tetravalent anions (*q* = −4) as shown in Fig. 6b. In the former case, the net pressure on the outer shell, shown as a function of the coating layer thickness, *R*_1_ − *R*_0_, in the figure, displays a relative change of only a few percent (around 5%) between the two cases with and without an ideally polarizable metallic core (“NP” vs “no NP”). In the case of multivalent anions, the effects of image charges are more drastic, changing the net-pressure profile from a monotonic to a non-monotonic one as shown in Fig. 6b. In this case, even at the largest coating layer thickness shown in the plot, *R*_1_ − *R*_0_ = 2 nm, the pressure decrease due to the image charges turns out to be about 2 atm in actual units, indicating that, in the process of encapsidating a metallic core, VLP stabilization can more significantly be assisted by the images of multivalent anions than those of multivalent cations. In both cases, the difference between “no NP” and “NP” cases decreases as *R*_1_ − *R*_0_ becomes comparable to or larger than the Debye screening length (here, ${\kappa }^{-1}\simeq 1.28$ nm).

To understand the difference in the image-charge effects found in the two cases mentioned above, one should first note that inserting (removing) the metallic NP core by itself can have the effect of decreasing (increasing) the net pressure, or making it more attractive (repulsive). This is due to the fact that, in the presence of a metallic core, the outer shell charge distribution, *σ*_2_, is pulled inward by its own oppositely charged image, being produced in the core (formally, this effect is embedded in Eq. (6)). This decrease in the net pressure, *P*, is independent of the choice of *q* and occurs equally in both cases shown in Fig. 6a,b. Therefore, the difference in the pressure drop in the two figures represents the intricate ways in which the pressure component due to multivalent ions, *P*_*q*_, is affected by the NP insertion. Again, one can generally expect to find a larger accumulation of multivalent ions inside the VLP upon the core insertion, which is driven by the attraction of multivalent ions and their (oppositely charged) images in the core; therefore, based only on their direct electrostatic interactions with the positive outer-shell charge distribution, one can anticipate a more positive *P*_*q*_ in the case of multivalent cations than in the case of multivalent anions. This rough argument however cannot explain the detailed features of the net-pressure profiles and misses other competing factors such as the additional counteracting attractive (repulsive) pressure that the image charges of multivalent cations (anions) exert on the outer shell, and the positive image charge of the coating layer, which appears upon the NP insertion in the VLP core, producing an additional positive pressure on the outer shell.

### Model VLP with and without NP core

**Figure 7:**
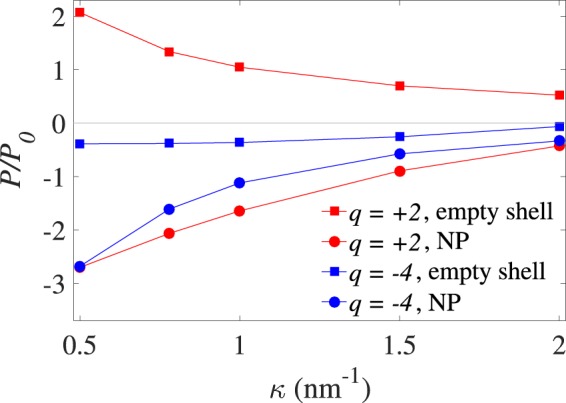
(Pressure vs *κ*). Rescaled net pressure plotted as a function of the inverse Debye screening length, *κ*, for *q* = + 2 and *q* = −4. We have compared the data obtained for a metallic-core VLP with those obtained for an empty shell (labelled “NP” and “empty shell” in the graph, respectively) with fixed *σ*_1_ = −1.5 nm^−2^. Other parameters are as in Fig. 2.

We proceed by comparing the results for a VLP containing a metallic NP (labeled by “NP”) with those obtained in an equivalent model in which the entire inside volume of the VLP is equally accessible to multivalent and monovalent (salt) ions, representing an “empty shell” model. In Fig. 7, the net pressure, *P*, on the outer shell is plotted as a function of the inverse Debye screening parameter, *κ*, for *q* = 2 and *q* = −4 (note that we have fixed *c*_0_ = 5 mM for divalent cations, and *c*_0_ = 1 mM for tetravalent anions; thus, as an example, *κ* = 2 nm^−1^ can be obtained by taking monovalent salt concentration of *n*_0_ = 376 mM in the former case and *n*_0_ = 379 mM in the latter case).

For the parameters given in Fig. 7, the net pressure *P* acting on the empty shell is positive, as it is dominated by the positive self-pressure, ${P}_{{\sigma }_{2}{\sigma }_{2}}$, of the shell in the case of divalent cations. By contrast, the net pressure becomes negative for tetravalent anions, due to the dominant negative *P*_*q*_, which is produced itself by the attractive strong-coupling interactions mediated by tetravalent anions between the opposing parts of the empty shell. This latter strong-coupling mechanism as engendered by multivalent counterions has been discussed in detail elsewhere^52^. This is also the reason why tetravalent anions produce the large negative pressure in region (I), especially for positive values of *σ*_1_, in Fig. 3a,b.

In both cases of *q* = 2 and *q* = −4 in Fig. 7, however, the net pressure on the outer shell becomes negative when a metallic NP core is inserted in the shell. This is an important observation, highlighting the substantial effect of the metallic NP core in decreasing the net pressure on the outer shell and making the VLP complex (electrostatically) significantly more stable than an empty virus-like shell.

## Conclusion

We study the role of electrostatic interactions in stabilization of a model virus-like particle (VLP) encapsidating a coated metallic nanoparticle (NP) in an asymmetric Coulomb fluid, which consists of a solution mixture of 1:1 and *q*:1 salts. Using Monte-Carlo (MC) simulations within an effective dressed multivalent-ion model^26,49–51^, we compute and analyze the net electrostatic or osmotic pressure acting on the outer VLP shell and identify the regimes of positive (outward-directed) and negative (inward-directed) net pressure in terms of multivalent cationic and anionic charge valencies, coating layer charge densities and thicknesses, and the bathing salt concentration. Without loss of generality, we assume that the outer shell of the VLP is positively charged and the sizes of the outer shell, the core particle and its coating layer are consistent with recent BMV experiments^9,15^. We elucidate the interplay between various cooperating or competing factors in the electrostatic stability of the VLP, such as the electrostatic self-pressure of the outer shell, the interaction between the outer shell and the coating layer charge (inner shell), the strong-coupling effects produced by multivalent (counter)ions, and the image charge effects that are produced by the ideally polarizable metallic NP core.

In the case of multivalent cations, an inward-directed net pressure on the outer shell, stabilizing the VLP, is found to occur only for negative coating layer charge densities of sufficiently large magnitude, which is generally consistent with recent experimental observations of coated, gold, NPs individually encapsulated within BMV capsids^15^. Multivalent anions can however generate negative net pressure on the outer shell for the whole range of positive and negative coating layer charge densities, with the pressure magnitude being of the order of few tens of atm. This suggests that multivalent anions can play a more robust role in electrostatic stabilization of VLP particles. Our analyses also show that the image charge effects, resulting from the insertion of a metallic NP, can generally make the VLP more stable (by reducing the net pressure on the outer shell or even by changing the sign of the pressure from positive to negative) as compared with an equivalent situation where the NP core is removed from the VLP. While the dominant mechanism at work for multivalent cations (and/or for sufficiently negative coating layer charge densities) is the inter-shell attraction between the positively charged outer shell and the negatively charged coating layer charge, the dominant mechanism in the case of multivalent anions turns out to be the inward-directed pressure they create due to their strong electrostatic coupling to the outer shell charge density. These effects have previously been addressed in detail in the case of empty shells or charge droplets^52,53^.

Our MC simulations are enabled by calculating the electrostatic interactions through the relevant Green’s function of the system (see the Supplementary Information), which consistently accounts for image charge effects (due to inhomogeneous dielectric constant and salt distribution in the system), salt screening effects and also strong electrostatic coupling effects due to multivalent ions that go beyond the scope of usual mean-field theories. Our dressed multivalent-ion implementation thus accounts for both the ionic screening effects due to the weakly coupled monovalent ions in the bathing solution and also the electrostatic correlations between multivalent ions and the opposite surface charges on the outer shell or the NP coating layer (see refs^22,23,26,27,49–53^ for further details).

The present model is constructed based on several simplifying assumptions, which, despite their limitations for the applicability to specific systems at full extent, posses two key advantages. First, they enable us to provide a thorough investigation of electrostatic effects that usually turn out to be very challenging because of the long-ranged nature of Coulomb interactions and the combined interplay between various factors such as mobile (multivalent) ions and image charge related effects^22,26,27^. As such, our model also helps circumventing difficulties in the computational implementation of the simulation within the present context. Secondly, our model can be used as a generic description for the NP encapsidation by a variety of proteinaceous shells in terms of a few basic parameters, whose numerical values can then be adopted according to the specific cases of interest.

Our model can be straightforwardly extended to include the different dielectric constants of the NP coating layer, that of the solution, and that of the NP core^60^. The more realistic aspects of viral capsids require more detailed modeling for the geometry and charge distribution of the capsid and the NP coating, stipulating more extensive coarse-grained or atomistic simulation techniques. Atomistic models can also help address the role of discrete nature of water molecules and its effect on the dielectric properties of the medium, especially inside the capsid. It is also worth mentioning that even models with the level of simplification we have used in this work^52,53^ would be able to capture the key electrostatic features of atomistic models; this is evidenced by the all-atom molecular-dynamics simulations of empty poliovirus capsids^61^, showing that the pressure acting on these empty capsids inside the solution can be negative due to electrostatic interactions, in accordance with our previous findings^52^.

Other factors that can be addressed in future studies include the role of specific-ion effects, detailed structure of multivalent ions^26,62,63^, charge regulation effects^64–66^, and (nonlinear) charge renormalization corrections^67,68^ to our linearized treatment of the electrostatic screening of the explicit charges by monovalent ions. In the present context, it should be noted that, even though the surface charge of the VLP in our simulations can appear to exceed the charge renormalization threshold predicted for charged (hard-sphere) colloids^67,68^, the presence of multivalent counterions in the system (accumulating strongly at and within the oppositely charged shells due to the permeability of the VLP structure) can drastically reduce the net surface potential experienced by the monovalent ions, minimizing and/or even eliminating possible charge renormalization of the VLP charge. Such effects are yet to be explored within the dressed multivalent-ion framework that forms the basis of our present study.

## Supplementary information

Supplementary Information

## Data Availability

The data generated and analyzed during this study are included in this published article, or are available from the corresponding author on reasonable requests.
