# Supplementary material for: Role of metallic core for the stability of virus-like particles in strongly coupled electrostatics
Source: Sci Rep. 2019 Mar 7;9:3884. doi: 10.1038/s41598-019-39930-8 (PMC6405863; doi:10.1038/s41598-019-39930-8)
Supplement: Supplementary file 1 — Supplementary Information [file 41598_2019_39930_MOESM1_ESM.pdf]

Electronic Supplementary Information: Role of metallic core for the stability of virus-like particles in strongly coupled electrostatics

Leili Javidpour,[1] Anže Lošdorfer Božič,[2] Rudolf Podgornik,[3,4,2,5] and Ali Naji[1]

[1]*School of Physics, Institute for Research in Fundamental Sciences (IPM), Tehran 19395-5531, Iran*
[2]*Department of Theoretical Physics, Jožef Stefan Institute, SI-1000 Ljubljana, Slovenia*
[3]*School of Physical Sciences and Kavli Institute for Theoretical Sciences, University of Chinese Academy of Sciences, Beijing 100049, China*
[4]*CAS Key Laboratory of Soft Matter Physics, Institute of Physics, Chinese Academy of Sciences (CAS), Beijing 100190, China*
[5]*Department of Physics, Faculty of Mathematics and Physics, University of Ljubljana, 1000 Ljubljana, Slovenia*

I. SCREENED COULOMB GREEN'S FUNCTION IN THE PRESENCE OF A METALLIC SPHERE

The Green's function $G(\mathbf{r},\mathbf{r}')$, describing electrostatic interactions of explicit charges within the dressed multivalent ion theory, is standardly obtained from the Debye-Hückel (DH) equations, governing the electrostatic potential in an electrolyte surrounding, in the present context, an ideally polarizable, metallic, nanoparticle (NP) of radius R_0 with constant surface (and interior) potential. Hence, by taking the center of coordinates at the center of the NP, we have

$$\begin{cases} G(\mathbf{r},\mathbf{r}') = C & , \ r \le R_0, \\ \nabla^2 G(\mathbf{r},\mathbf{r}') - \kappa^2 G(\mathbf{r},\mathbf{r}') = -\frac{1}{\varepsilon\varepsilon_0}\delta(\mathbf{r}-\mathbf{r}') & , \ r > R_0, \end{cases} \tag{1}$$

where C is a constant. The solution to the above set of equations in the region outside the spherical NP can be expressed as the sum of a "special" solution (first term below), representing the bulk solution $G_0(\mathbf{r},\mathbf{r}') = \mathrm{e}^{-\kappa|\mathbf{r}-\mathbf{r}'|}/(4\pi\varepsilon\varepsilon_0|\mathbf{r}-\mathbf{r}'|)$, and a "homogenous" solution (second term below) due to the presence of the NP [1],

$$G(\mathbf{r},\mathbf{r}') = \frac{1}{4\pi\varepsilon\varepsilon_0}\frac{\exp(-\kappa|\mathbf{r}-\mathbf{r}'|)}{|\mathbf{r}-\mathbf{r}'|} + \sum_{l=0}^{\infty} B_l k_l(\kappa r) P_l(\cos\vartheta), \tag{2}$$

where $k_l(\cdot)$ are modified spherical Bessel functions of the second kind, $P_l(\cdot)$ are Legendre polynomials, and we have defined $r = |\mathbf{r}|$, $r' = |\mathbf{r}'|$, and ϑ as the angle between $\mathbf{r}$ and $\mathbf{r}'$. The coefficients B_l are in general functions of r'. The first term above can be expanded as [1]

$$\frac{\exp(-\kappa|\mathbf{r}-\mathbf{r}'|)}{|\mathbf{r}-\mathbf{r}'|} = \kappa\sum_{l=0}^{\infty}(2l+1) i_l(\kappa r_<) k_l(\kappa r_>) P_l(\cos\vartheta), \tag{3}$$

in which $i_l(\cdot)$ are modified spherical Bessel functions of the first kind and $r_<$ and $r_>$ denote the smaller and larger values of r and r'. Since the potential on the metallic sphere is constant and does not depend on ϑ, and using $r_< = r = R_0$ and $r_> = r'$, we find

$$\begin{aligned} C &= B_0 k_0(\kappa R_0) + \tfrac{\kappa}{4\pi\varepsilon\varepsilon_0} i_0(\kappa R_0) k_0(\kappa r'), && \text{for } l = 0, \\ 0 &= B_l k_l(\kappa R_0) + \tfrac{\kappa}{4\pi\varepsilon\varepsilon_0}(2l+1) i_l(\kappa R_0) k_l(\kappa r'), && \text{for } l > 0. \end{aligned} \tag{4}$$

and, hence,

$$\begin{aligned} B_0 &= \tfrac{C}{k_0(\kappa R_0)} - \tfrac{\kappa}{4\pi\varepsilon\varepsilon_0}\tfrac{i_0(\kappa R_0)}{k_0(\kappa R_0)} k_0(\kappa r'), \\ B_l &= -\tfrac{\kappa}{4\pi\varepsilon\varepsilon_0}(2l+1)\tfrac{i_l(\kappa R_0)}{k_l(\kappa R_0)} k_l(\kappa r'), \end{aligned} \tag{5}$$

which give the solution in the outside region, $r, r' \ge R_0$, as

$$G(\mathbf{r},\mathbf{r}') = C\frac{k_0(\kappa r)}{k_0(\kappa R_0)} - \frac{\kappa}{4\pi\varepsilon\varepsilon_0}\sum_{l=0}^{\infty}(2l+1)\frac{i_l(\kappa R_0)}{k_l(\kappa R_0)} k_l(\kappa r') k_l(\kappa r) P_l(\cos\vartheta) + \frac{1}{4\pi\varepsilon\varepsilon_0}\frac{\exp(-\kappa|\mathbf{r}-\mathbf{r}'|)}{|\mathbf{r}-\mathbf{r}'|}. \tag{6}$$

The constant C can be fixed by using the fact that the metallic NP is assumed to be electroneutral; hence, using Gauss's law and after straightforward manipulations, we find

$$C = \frac{\kappa}{4\pi\varepsilon\varepsilon_0}\frac{k_0(\kappa r')}{k_0'(\kappa R_0)}\left(i_0(\kappa R_0) k_0'(\kappa R_0) - i_0'(\kappa R_0) k_0(\kappa R_0)\right) = \frac{1}{4\pi\varepsilon\varepsilon_0}\frac{\mathrm{e}^{-\kappa(r'-R_0)}}{r'(1+\kappa R_0)}, \tag{7}$$

where we have used the explicit expressions

$$i_0(x) = \frac{\sinh x}{x}, \quad i_0'(x) = \frac{x\cosh x - \sinh x}{x^2}, \quad k_0(x) = \frac{\mathrm{e}^{-x}}{x}, \quad k_0'(x) = -\frac{(1+x)\mathrm{e}^{-x}}{x^2}. \tag{8}$$

The final expression for the Green's function can thus be obtained as

$$G(\mathbf{r},\mathbf{r}') = G_0(\mathbf{r},\mathbf{r}') + G_{im}(\mathbf{r},\mathbf{r}'), \tag{9}$$

where $G_{im}(\mathbf{r},\mathbf{r}')$ is the contribution representing salt/dielectric image effects,

$$G_{im}(\mathbf{r},\mathbf{r}') = -\frac{\kappa}{4\pi\varepsilon\varepsilon_0}\sum_{l=0}^{\infty}(2l+1)\frac{i_l(\kappa R_0)}{k_l(\kappa R_0)} k_l(\kappa r) k_l(\kappa r') P_l(\cos\vartheta) + \frac{\kappa^2 R_0\,\mathrm{e}^{2\kappa R_0}}{4\pi\varepsilon\varepsilon_0(1+\kappa R_0)} k_0(\kappa r) k_0(\kappa r'). \tag{10}$$

II. HAMILTONIAN OF THE MODEL VLP

In the VLP model used in the main text, the charge distribution of the inner and outer spherical shells (of radii R_1 and R_2) can formally be expressed as

$$\rho_\sigma(\mathbf{r}) = \sum_{\alpha=1}^{2} \sigma_\alpha e_0 \delta(r - R_\alpha). \tag{11}$$

Other explicit charges in the system include multivalent ions each of charge valency q, located at positions $\{\mathbf{r}_i\}$, giving the local charge distribution function

$$\rho(\mathbf{r}) = \sum_{i=1}^{N} q e_0 \delta(\mathbf{r} - \mathbf{r}_i). \tag{12}$$

The Hamiltonian associated with electrostatic interactions in the system can in general be written as

$$H = \frac{1}{2}\sum_{i,j=1}^{N} q^2 e_0^2 G(\mathbf{r}_i, \mathbf{r}_j) + \sum_{i=1}^{N} q e_0 \int \mathrm{d}\mathbf{r} \rho_\sigma(\mathbf{r}) G(\mathbf{r}, \mathbf{r}_i) + \frac{1}{2}\int \mathrm{d}\mathbf{r}\, \mathrm{d}\mathbf{r}'\, \rho_\sigma(\mathbf{r}) G(\mathbf{r}, \mathbf{r}') \rho_\sigma(\mathbf{r}'). \tag{13}$$

Let us first focus on the case of only one multivalent ion in the system positioned at $\mathbf{b}$ (note that multivalent ion positions are restricted to remain outside the inner shell, i.e., $b = |\mathbf{b}| > R_1$). We will thus have

$$H = \frac{q^2 e_0^2}{2} G(\mathbf{b}, \mathbf{b}) + q e_0 \int \mathrm{d}\mathbf{r} \rho_\sigma(\mathbf{r}) G(\mathbf{r}, \mathbf{b}) + \frac{1}{2}\int \mathrm{d}\mathbf{r}\, \mathrm{d}\mathbf{r}'\, \rho_\sigma(\mathbf{r}) G(\mathbf{r}, \mathbf{r}') \rho_\sigma(\mathbf{r}') \equiv H_{im} + H_\sigma + H_{\sigma\sigma}. \tag{14}$$

The first term in Eq. (14) is the self-energy of the multivalent ion and its image interaction. We subtract the redundant (infinite) vacuum self-energy of the multivalent ion, and the ion-image interaction term is found as

$$H_{im} = -\frac{q^2 e_0^2 \kappa}{8\pi\varepsilon\varepsilon_0} \sum_{l=0}^{\infty} (2l+1) \frac{i_l(\kappa R_0)}{k_l(\kappa R_0)} k_l^2(\kappa b) + \frac{q^2 e_0^2 \kappa^2 R_0\, \mathrm{e}^{2\kappa R_0}}{8\pi\varepsilon\varepsilon_0 (1 + \kappa R_0)} k_0^2(\kappa b). \tag{15}$$

The second term in Eq. (14) is the interaction between the ion and the surface charge, including both the direct DH and the image interactions. For the α-th shell, it yields

$$H_\sigma = \sum_{\alpha=1}^{2} q\sigma_\alpha e_0^2 \int \mathrm{d}\mathbf{r}\, \delta(r - R_\alpha) G(\mathbf{r}, \mathbf{b}) = \sum_{\alpha=1}^{2} q\sigma_\alpha e_0^2 \int r^2 \mathrm{d}r\, \mathrm{d}\Omega\, \delta(r - R_\alpha) [G_0(\mathbf{r}, \mathbf{b}) + G_{im}(\mathbf{r}, \mathbf{b})] \equiv H_\sigma^{dir} + H_\sigma^{im}. \tag{16}$$

The direct interaction is

$$\begin{aligned} H_\sigma^{dir} &= \sum_{\alpha=1}^{2} q\sigma_\alpha e_0^2 \int r^2 \mathrm{d}r\, \mathrm{d}\Omega\, \delta(r - R_\alpha) G_0(\mathbf{r}, \mathbf{b}) \\ &= \sum_{\alpha=1}^{2} \frac{q\sigma_\alpha e_0^2}{4\pi\varepsilon\varepsilon_0} \int r^2 \mathrm{d}r\, \mathrm{d}\Omega\, \delta(r - R_\alpha) \frac{\mathrm{e}^{-\kappa|\mathbf{r}-\mathbf{b}|}}{|\mathbf{r}-\mathbf{b}|} = \sum_{\alpha=1}^{2} \frac{q\sigma_\alpha e_0^2 R_\alpha^2}{4\pi\varepsilon\varepsilon_0} \int \mathrm{d}\Omega\, \frac{\mathrm{e}^{-\kappa\sqrt{R_\alpha^2 + b^2 - 2R_\alpha b\cos\vartheta}}}{\sqrt{R_\alpha^2 + b^2 - 2R_\alpha b\cos\vartheta}}, \end{aligned} \tag{17}$$

where ϑ is the angle between $\mathbf{r}$ and $\mathbf{b}$. The direct interaction term can be evaluated as

$$H_\sigma^{dir} = \sum_{\alpha=1}^{2} \frac{q\sigma_\alpha e_0^2 R_\alpha^2}{2\varepsilon\varepsilon_0} \int_{-1}^{1} \mathrm{d}u \frac{\mathrm{e}^{-\kappa\sqrt{R_\alpha^2 + b^2 - 2R_\alpha b u}}}{\sqrt{R_\alpha^2 + b^2 - 2R_\alpha b u}} = \sum_{\alpha=1}^{2} \frac{q\sigma_\alpha e_0^2 R_\alpha}{2\kappa b\varepsilon\varepsilon_0} \left(\mathrm{e}^{-\kappa|b - R_\alpha|} - \mathrm{e}^{-\kappa|b + R_\alpha|} \right). \tag{18}$$

The image interaction part, on the other hand, is obtained as

$$\begin{aligned} H_\sigma^{im} &= \sum_{\alpha=1}^{2} q\sigma_\alpha e_0^2 \int r^2 \mathrm{d}r\, \mathrm{d}\Omega\; \delta(r - R_\alpha) G_{im}(\mathbf{r}, \mathbf{b}) \\ &= -\sum_{\alpha=1}^{2} \frac{\kappa q\sigma_\alpha e_0^2 R_\alpha^2}{4\pi\varepsilon\varepsilon_0} \int \mathrm{d}\Omega \left(\sum_{l=0}^{\infty} (2l+1) \frac{i_l(\kappa R_0)}{k_l(\kappa R_0)} k_l(\kappa R_\alpha) k_l(\kappa b) P_l(\cos\vartheta) - \frac{\kappa R_0\, \mathrm{e}^{2\kappa R_0}}{1 + \kappa R_0} k_0(\kappa R_\alpha) k_0(\kappa b) \right). \end{aligned} \tag{19}$$

The integral over the Legendre functions is non-zero only for $l = 0$, leaving us with

$$H_\sigma^{im} = -\sum_{\alpha=1}^{2} \frac{\kappa q \sigma_\alpha e_0^2 R_\alpha^2}{\varepsilon\varepsilon_0} \left(\frac{i_0(\kappa R_0)}{k_0(\kappa R_0)} k_0(\kappa R_\alpha) k_0(\kappa b) - \frac{\kappa R_0\, \mathrm{e}^{2\kappa R_0}}{1+\kappa R_0} k_0(\kappa R_\alpha) k_0(\kappa b) \right)$$
$$= -\sum_{\alpha=1}^{2} \frac{\kappa q \sigma_\alpha e_0^2 R_\alpha^2}{\varepsilon\varepsilon_0} \left(\mathrm{e}^{\kappa R_0} \sinh \kappa R_0 \frac{\mathrm{e}^{-\kappa R_\alpha}}{\kappa R_\alpha} \frac{\mathrm{e}^{-\kappa b}}{\kappa b} - \frac{\kappa R_0\, \mathrm{e}^{2\kappa R_0}}{1+\kappa R_0} \frac{\mathrm{e}^{-\kappa R_\alpha}}{\kappa R_\alpha} \frac{\mathrm{e}^{-\kappa b}}{\kappa b} \right). \tag{20}$$

The net contribution from the second term in Eq. (14) is thus obtained as

$$H_\sigma = \sum_{\alpha=1}^{2} \left[\frac{q\sigma_\alpha e_0^2 R_\alpha}{2\kappa b \varepsilon\varepsilon_0} \left(\mathrm{e}^{-\kappa|b-R_\alpha|} - \mathrm{e}^{-\kappa(b+R_\alpha)} \right) + \frac{q\sigma_\alpha e_0^2 R_\alpha}{2\kappa b \varepsilon\varepsilon_0} \mathrm{e}^{-\kappa(b+R_\alpha)} \left(1 + \mathrm{e}^{2\kappa R_0} \frac{\kappa R_0 - 1}{\kappa R_0 + 1} \right) \right]. \tag{21}$$

For the last part of Eq. (14), which gives the contribution from surface-surface interaction (including the relevant image effects), we can write

$$H_{\sigma\sigma} = \sum_{\alpha,\beta=1}^{2} \frac{e_0^2}{2} \int \mathrm{d}\mathbf{r}\, \mathrm{d}\mathbf{r}' \sigma_\alpha \delta(r-R_\alpha)[G_0(\mathbf{r},\mathbf{r}') + G_{im}(\mathbf{r},\mathbf{r}')]\sigma_\beta \delta(r'-R_\beta) \equiv H_{\sigma\sigma}^{dir} + H_{\sigma\sigma}^{im}. \tag{22}$$

The direct interaction part here is given by

$$H_{\sigma\sigma}^{dir} = \sum_{\alpha,\beta=1}^{2} \frac{e_0^2}{2} \int \mathrm{d}\mathbf{r}\, \mathrm{d}\mathbf{r}' \sigma_\alpha \delta(r-R_\alpha) G_0(\mathbf{r},\mathbf{r}') \sigma_\beta \delta(r'-R_\beta), \tag{23}$$

or,

$$H_{\sigma\sigma}^{dir} = \sum_{\alpha,\beta=1}^{2} \frac{\sigma_\alpha \sigma_\beta e_0^2}{2} \int r^2 \mathrm{d}r\, \mathrm{d}\Omega\, r'^2 \mathrm{d}r' \mathrm{d}\Omega'\, \delta(r-R_\alpha)\delta(r'-R_\beta) \frac{\mathrm{e}^{-\kappa|\mathbf{r}-\mathbf{r}'|}}{4\pi\varepsilon\varepsilon_0 |\mathbf{r}-\mathbf{r}'|}, \tag{24}$$

giving

$$H_{\sigma\sigma}^{dir} = \sum_{\alpha,\beta=1}^{2} \frac{\sigma_\alpha \sigma_\beta e_0^2 R_\alpha^2 R_\beta^2}{8\pi\varepsilon\varepsilon_0} \int \mathrm{d}\Omega'\, \mathrm{d}\Omega\, \frac{\mathrm{e}^{-\kappa\sqrt{R_\alpha^2 + R_\beta^2 - 2R_\alpha R_\beta \cos\vartheta}}}{\sqrt{R_\alpha^2 + R_\beta^2 - 2R_\alpha R_\beta \cos\vartheta}}. \tag{25}$$

The first angular integration above can be done straightforwardly, and since the result is independent of the angle between the two vectors, the second angular integration only yields a constant. Thus,

$$H_{\sigma\sigma}^{dir} = \sum_{\alpha,\beta=1}^{2} \frac{\sigma_\alpha \sigma_\beta e_0^2 R_\alpha^2 R_\beta^2}{8\pi\varepsilon\varepsilon_0} 4\pi \frac{2\pi}{\kappa R_\alpha R_\beta} \left(\mathrm{e}^{-\kappa|R_\alpha - R_\beta|} - \mathrm{e}^{-\kappa(R_\alpha+R_\beta)} \right) = \sum_{\alpha,\beta=1}^{2} \frac{\pi \sigma_\alpha \sigma_\beta e_0^2 R_\alpha R_\beta}{\kappa\varepsilon\varepsilon_0} \left(\mathrm{e}^{-\kappa|R_\alpha - R_\beta|} - \mathrm{e}^{-\kappa(R_\alpha+R_\beta)} \right). \tag{26}$$

The image interaction part, on the other hand, is obtained as

$$H_{\sigma\sigma}^{im} = \sum_{\alpha,\beta=1}^{2} \frac{e_0^2}{2} \int \mathrm{d}\mathbf{r}\, \mathrm{d}\mathbf{r}' \sigma_\alpha \delta(r-R_\alpha) G_{im}(\mathbf{r},\mathbf{r}') \sigma_\beta \delta(r'-R_\beta), \tag{27}$$

or, similarly as before,

$$H_{\sigma\sigma}^{im} = -\sum_{\alpha,\beta=1}^{2} \frac{\kappa \sigma_\alpha \sigma_\beta e_0^2 R_\alpha^2 R_\beta^2}{8\pi\varepsilon\varepsilon_0}$$
$$\times \left(\sum_l (2l+1) \frac{i_l(\kappa R_0)}{k_l(\kappa R_0)} k_l(\kappa R_\alpha) k_l(\kappa R_\beta) \int \mathrm{d}\Omega'\, \mathrm{d}\Omega\, P_l(\cos\vartheta) - \int \mathrm{d}\Omega'\, \mathrm{d}\Omega\, \frac{\kappa R_0\, \mathrm{e}^{2\kappa R_0}}{1+\kappa R_0} k_0(\kappa R_\alpha) k_0(\kappa R_\beta) \right),$$

from which we obtain

$$H^{im}_{\sigma\sigma} = \sum_{\alpha,\beta=1}^{2} -\frac{2\pi\kappa\sigma_\alpha\sigma_\beta e_0^2 R_\alpha^2 R_\beta^2}{\varepsilon\varepsilon_0}\left(\frac{i_0(\kappa R_0)}{k_0(\kappa R_0)}k_0(\kappa R_\alpha)k_0(\kappa R_\beta) - \frac{\kappa R_0\,\mathrm{e}^{2\kappa R_0}}{1+\kappa R_0}k_0(\kappa R_\alpha)k_0(\kappa R_\beta)\right)$$
$$= \sum_{\alpha,\beta=1}^{2}\frac{\pi\sigma_\alpha\sigma_\beta e_0^2 R_\alpha R_\beta}{\kappa\varepsilon\varepsilon_0}\left(1+\mathrm{e}^{2\kappa R_0}\frac{\kappa R_0-1}{\kappa R_0+1}\right)\mathrm{e}^{-\kappa(R_\alpha+R_\beta)}. \tag{28}$$

Hence, we have

$$H_{\sigma\sigma} = \sum_{\alpha,\beta=1}^{2}\frac{\pi\sigma_\alpha\sigma_\beta e_0^2 R_\alpha R_\beta}{\kappa\varepsilon\varepsilon_0}\left(\mathrm{e}^{-\kappa|R_\alpha-R_\beta|}-\mathrm{e}^{-\kappa(R_\alpha+R_\beta)}\right)+\sum_{\alpha,\beta=1}^{2}\frac{\pi\sigma_\alpha\sigma_\beta e_0^2 R_\alpha R_\beta}{\kappa\varepsilon\varepsilon_0}\left(1+\mathrm{e}^{2\kappa R_0}\frac{\kappa R_0-1}{\kappa R_0+1}\right)\mathrm{e}^{-\kappa(R_\alpha+R_\beta)}. \tag{29}$$

Putting the three terms contributing to the Hamiltonian together, i.e., $H = H_{im} + H_\sigma + H_{\sigma\sigma}$, we have

$$H = -\frac{\kappa q^2 e_0^2}{8\pi\varepsilon\varepsilon_0}\sum_{l=0}^{\infty}(2l+1)\frac{i_l(\kappa R_0)}{k_l(\kappa R_0)}k_l^2(\kappa b) + \frac{\kappa^2 R_0 q^2 e_0^2\,\mathrm{e}^{2\kappa R_0}}{8\pi\varepsilon\varepsilon_0(1+\kappa R_0)}k_0^2(\kappa b)$$
$$+\frac{qe_0^2}{2\kappa b\varepsilon\varepsilon_0}\sum_{\alpha=1}^{2}\sigma_\alpha R_\alpha\left[\left(\mathrm{e}^{-\kappa|b-R_\alpha|}-\mathrm{e}^{-\kappa(b+R_\alpha)}\right)+\mathrm{e}^{-\kappa(b+R_\alpha)}\left(1+\mathrm{e}^{2\kappa R_0}\frac{\kappa R_0-1}{\kappa R_0+1}\right)\right]$$
$$+\frac{\pi e_0^2}{\kappa\varepsilon\varepsilon_0}\sum_{\alpha,\beta=1}^{2}\sigma_\alpha\sigma_\beta R_\alpha R_\beta\left[\left(\mathrm{e}^{-\kappa|R_\alpha-R_\beta|}-\mathrm{e}^{-\kappa(R_\alpha+R_\beta)}\right)+\mathrm{e}^{-\kappa(R_\alpha+R_\beta)}\left(1+\mathrm{e}^{2\kappa R_0}\frac{\kappa R_0-1}{\kappa R_0+1}\right)\right]. \tag{30}$$

Now, when we have N multivalent ions in the system, the Hamiltonian can straightforwardly be expressed as

$$H = -\sum_{i=1}^{N}\frac{\kappa q^2 e_0^2}{8\pi\varepsilon\varepsilon_0}\sum_{l=0}^{\infty}(2l+1)\frac{i_l(\kappa R_0)}{k_l(\kappa R_0)}k_l^2(\kappa r_i)+\sum_{i=1}^{N}\frac{\kappa^2 R_0 q^2 e_0^2\,\mathrm{e}^{2\kappa R_0}}{8\pi\varepsilon\varepsilon_0(1+\kappa R_0)}k_0^2(\kappa r_i)$$
$$+\sum_{i=1}^{N}\frac{qe_0^2}{2\kappa r_i\varepsilon\varepsilon_0}\sum_{\alpha=1}^{2}\sigma_\alpha R_\alpha\left[\left(\mathrm{e}^{-\kappa|r_i-R_\alpha|}-\mathrm{e}^{-\kappa(r_i+R_\alpha)}\right)+\mathrm{e}^{-\kappa(r_i+R_\alpha)}\left(1+\mathrm{e}^{2\kappa R_0}\frac{\kappa R_0-1}{\kappa R_0+1}\right)\right]$$
$$+\frac{\pi e_0^2}{\kappa\varepsilon\varepsilon_0}\sum_{\alpha,\beta=1}^{2}\sigma_\alpha\sigma_\beta R_\alpha R_\beta\left[\left(\mathrm{e}^{-\kappa|R_\alpha-R_\beta|}-\mathrm{e}^{-\kappa(R_\alpha+R_\beta)}\right)+\mathrm{e}^{-\kappa(R_\alpha+R_\beta)}\left(1+\mathrm{e}^{2\kappa R_0}\frac{\kappa R_0-1}{\kappa R_0+1}\right)\right]$$
$$+\sum_{i>j=1}^{N}\frac{q^2 e_0^2}{4\pi\varepsilon\varepsilon_0}\frac{\mathrm{e}^{-\kappa|\mathbf{r}_i-\mathbf{r}_j|}}{|\mathbf{r}_i-\mathbf{r}_j|}$$
$$-\sum_{i>j=1}^{N}\frac{\kappa q^2 e_0^2}{4\pi\varepsilon\varepsilon_0}\sum_{l=0}^{\infty}(2l+1)\frac{i_l(\kappa R_0)}{k_l(\kappa R_0)}k_l(\kappa r_i)k_l(\kappa r_j)P_l(\cos\vartheta)+\sum_{i>j=1}^{N}\frac{\kappa^2 R_0 q^2 e_0^2\,\mathrm{e}^{2\kappa R_0}}{4\pi\varepsilon\varepsilon_0(1+\kappa R_0)}k_0(\kappa r_i)k_0(\kappa r_j). \tag{31}$$

This completes the derivation of the expressions given in Eqs. (2)-(6) of the main text.

III. NET PRESSURE ON THE OUTER SHELL

In the *absence* of a metallic core within the VLP, the net electrostatic potential of the two charged shells with radii R_1 and R_2 is obtained as

$$\varphi_1(0\le r\le R_1) = \frac{e_0}{\varepsilon\varepsilon_0}\left(\sigma_1\mathrm{e}^{-\kappa R_1}R_1+\sigma_2\mathrm{e}^{-\kappa R_2}R_2\right)\frac{\sinh\kappa r}{\kappa r}, \tag{32}$$

$$\varphi_2(R_1< r\le R_2) = \frac{e_0}{\varepsilon\varepsilon_0}\left[\left(\sigma_2\mathrm{e}^{-\kappa R_2}R_2\right)\frac{\sinh\kappa r}{\kappa r}+\left(\sigma_1 R_1\sinh\kappa R_1\right)\frac{\mathrm{e}^{-\kappa r}}{\kappa r}\right], \tag{33}$$

$$\varphi_3(r>R_2) = \frac{e_0}{\varepsilon\varepsilon_0}\left(\sigma_1 R_1\sinh\kappa R_1+\sigma_2 R_2\sinh\kappa R_2\right)\frac{\mathrm{e}^{-\kappa r}}{\kappa r}. \tag{34}$$

The free energy of the system in the absence of multivalent ions then follows standardly as

$$F_{DH} = \frac{\pi e_0^2}{\kappa\varepsilon\varepsilon_0}\left\{\sigma_1^2 R_1^2(1-\mathrm{e}^{-2\kappa R_1}) + \sigma_2^2 R_2^2(1-\mathrm{e}^{-2\kappa R_2}) + 2\sigma_1\sigma_2 R_1 R_2(\mathrm{e}^{-\kappa(R_2-R_1)} - \mathrm{e}^{-\kappa(R_1+R_2)})\right\}. \tag{35}$$

The corresponding net (osmotic) pressure acting on the outer shell follows from

$$P_{DH} = -\left.\frac{\partial F_{DH}}{\partial V_2}\right|_{Q_2}, \tag{36}$$

where $V_2 = 4\pi R_2^3/3$ is the volume of the outer shell and the partial derivative is taken at fixed value of the total surface charge of this shell, i.e., $Q_2 = 4\pi R_2^2\sigma_2$. We thus find

$$P_{DH} = \frac{\sigma_2^2 e_0^2}{2\varepsilon\varepsilon_0}\left\{\frac{1}{\kappa R_2} - \mathrm{e}^{-2\kappa R_2}\left(1+\frac{1}{\kappa R_2}\right)\right\} + \frac{\sigma_1\sigma_2 e_0^2}{2\varepsilon\varepsilon_0}\frac{R_1}{R_2}\left\{\mathrm{e}^{-\kappa(R_2-R_1)} - \mathrm{e}^{-\kappa(R_1+R_2)}\right\}\left(1+\frac{1}{\kappa R_2}\right). \tag{37}$$

The contribution of multivalent ions to the osmotic pressure follows as (see Refs. [2, 3])

$$P_q = -\left\langle \sum_{i=1}^{N} q e_0 \left.\frac{\partial\varphi(\mathbf{r}_i)}{\partial V_2}\right|_{Q_2}\right\rangle, \tag{38}$$

where the potential φ is defined in piece-wise fashion throughout the space according to expressions (32)-(34). Since multivalent ions are restricted to remain outside the inner shell $|\mathbf{r}_i| > R_1$, we shall only require

$$\left.\frac{\partial\varphi_2(R_1 < r \le R_2)}{\partial V_2}\right|_{Q_2} = \frac{\sigma_2 e_0 \sinh\kappa r}{4\pi\varepsilon\varepsilon_0\kappa r R_2^2}(1+\kappa R_2)\mathrm{e}^{-\kappa R_2}, \tag{39}$$

and

$$\left.\frac{\partial\varphi_3(r > R_2)}{\partial V_2}\right|_{Q_2} = \frac{\sigma_2 e_0 \mathrm{e}^{-\kappa r}}{4\pi\varepsilon\varepsilon_0\kappa r R_2^2}(\kappa R_2\cosh\kappa R_2 - \sinh\kappa R_2). \tag{40}$$

In the *presence* of a metallic core within the VLP, the potential derivative can be obtained from the second term in Eq. (30) as

$$\left.\frac{\partial\varphi(\mathbf{r}_i)}{\partial V_2}\right|_{Q_2} = \frac{\sigma_2 e_0}{8\pi\varepsilon\varepsilon_0\kappa R_2^2}\sum_{i=1}^{N}\left[\frac{\mathrm{e}^{-\kappa|r_i-R_2|}}{r_i}(\kappa R_2\,\mathrm{sgn}(r_i-R_2)-1) - \frac{\mathrm{e}^{-\kappa(r_i+R_2-2R_0)}}{r_i}(1+\kappa R_2)\left(\frac{\kappa R_0-1}{\kappa R_0+1}\right)\right]. \tag{41}$$

This expression can be used to construct the contribution of multivalent ions to the osmotic pressure, as expressed in Eqs. (12) and (13) in the main text. Also, the third term in Eq. (30), can be used to obtain Eqs. (9)-(11) in the main text.

IV. ITERATIVE CANONICAL ALGORITHM

As noted in the main text, the ionic bulk due to the explicit multivalent ions is established in our simulations using the iterative canonical MC algorithm introduced by us in Refs. [2, 3], while the ionic bulk for monovalent ions is implicitly assumed. The mentioned method utilizes a series of individual (full) canonical simulations that are performed iteratively for any given set of parameter values and for a prescribed value of the bulk concentration, c_0, for the multivalent ions. It is assumed that the number of explicit multivalent ions placed in the simulation box and the bulk concentration obtained for them through the simulations are monotonically related. This latter assumption is numerically verified. The goal is then to design the simulations such that, in consecutive steps i and $i+1$ of the iterations, the resulting (simulated) bulk concentrations c_i and c_{i+1} bracket the prescribed value c_0 with increasingly improved accuracy; hence, the corresponding numbers of multivalent ions N_i and N_{i+1} used in the simulations, which are estimated based on a linear interpolation scheme and based on the information from the previous steps of the iterations (see below), bracket and converge to the desired value that produces the prescribed value of the bulk concentration, c_0. The bulk concentration obtained in each individual simulation is read off from the equilibrium plateau-like region of the simulated density profile of multivalent ions, as established within the simulation box and at sufficiently large distances away from the central VLP. We have verified that the proposed simulation cycle always converges to the desired limit for each data set reported in the main text.

The algorithm can be summarized in practical terms as follows.

- Initialization:
  - ▷ Set up an initial simulation with the number of multivalent ions being set equal to $N_{\rm ini} = N_0 + C_N$, where C_N and N_0 are chosen for practical convenience as $C_N = 10$ and $N_0 = \max\{1, c_0 V_b - (Q_1 + Q_2)/q\}$ (these choices are of no physical significance for the outcomes). Here, $c_0 V_b$ is the number of multivalent ions in the simulation box of volume V_b, if they were to be distributed evenly within the box, and $-(Q_1 + Q_2)/q$ is the excess number of multivalent ions required to compensate the sum of the fixed charges on the two shells $Q_\alpha = 4\pi\sigma_\alpha R_\alpha^2$, where $\alpha = 1$ and 2 for the inner and outer shells, respectively.
  - ▷ Run a full simulation using $N_{\rm ini}$ multivalent ions and obtain the resulting bulk concentration $c_{\rm ini}$.
  - ▷ If $c_{\rm ini} < c_0$, re-run the initial simulation by setting $N_{\rm ini} \to N_{\rm ini} + C_N$ and repeat as necessary until a situation with $c_{\rm ini} > c_0$ is reached.
  - ▷ The initial bracketing of the prescribed c_0 is thus achieved by storing the last values of $N_{\rm ini}$ and $c_{\rm ini}$ obtained through the preceding steps as the upper-bound values $N_{\rm max} = N_{\rm ini}$ and $c_{\rm max} = c_{\rm ini}$, and by setting the lower-bound values as $N_{\rm min} = 1$ and $c_{\rm min} = 0$; the latter are reasonable lower-bound choices, as with just one multivalent ion, our simulations give a nearly vanishing bulk concentration.
- Interpolation and iteration:
  - ▷ Set $N_* = (N_{\rm max} - N_{\rm min})(c_0 - c_{\rm min})/(c_{\rm max} - c_{\rm min}) + N_{\rm min}$.
  - ▷ Run a full simulation using N_* multivalent ions and obtain the resulting bulk concentration c_*.
  - ▷ If $c_* > c_0$, store N_* and c_* as the new upper-bound values ($N_{\rm max} = N_*$, $c_{\rm max} = c_*$), while the lower-bound values are carried on from the previous step;
  - ▷ Otherwise, store N_* and c_* as the new lower-bound values ($N_{\rm min} = N_*$, $c_{\rm min} = c_*$), while the upper-bound values are carried on from the previous step.
  - ▷ Repeat the above four steps until $|c_* - c_0| < \epsilon\, c_0$ is satisfied, where the convergence relative error is conventionally taken as $\epsilon = 10^{-3}$.
- The data from the final simulation achieved through the above iterative steps produce the desirable outcomes.

---

[1] G. Arfken, *Mathematical Methods for Physicists*, Third Edition (Academic Press, Inc. 1985).
[2] L. Javidpour, A. Lošdorfer Božič, A. Naji and R. Podgornik, J. Chem. Phys. **139**, 154709 (2013).
[3] L. Javidpour, A. Lošdorfer Božič, A. Naji and R. Podgornik, Soft Matter **9**, 11357 (2013).
